# Reprogramming of Sheep Fibroblasts into Pluripotency under a Drug-Inducible Expression of Mouse-Derived Defined Factors

**DOI:** 10.1371/journal.pone.0015947

**Published:** 2011-01-06

**Authors:** Yang Li, Ming Cang, Andrew Stephen Lee, Kehua Zhang, Dongjun Liu

**Affiliations:** 1 Stem Cell Research Center, Peking University Health Science Center, Beijing, China; 2 Lab Animal Research Center, College of Life Sciences, Inner Mongolia University, Huhhot, China; 3 Division of Cardiology, Department of Medicine, Stanford University School of Medicine, Stanford, California, United States of America; 4 Department of Immunity, Clinical Medicine Institute, China Japan Friendship Hospital, Beijing, China; 5 Department of Cell Biology, Peking University Health Science Center, Beijing, China; University of Minnesota, United States of America

## Abstract

Animal embryonic stem cells (ESCs) provide powerful tool for studies of early embryonic development, gene targeting, cloning, and regenerative medicine. However, the majority of attempts to establish ESC lines from large animals, especially ungulate mammals have failed. Recently, another type of pluripotent stem cells, known as induced pluripotent stem cells (iPSCs), have been successfully generated from mouse, human, monkey, rat and pig. In this study we show sheep fibroblasts can be reprogrammed to pluripotency by defined factors using a drug-inducible system. Sheep iPSCs derived in this fashion have a normal karyotype, exhibit morphological features similar to those of human ESCs and express AP, Oct4, Sox2, Nanog and the cell surface marker SSEA-4. Pluripotency of these cells was further confirmed by embryoid body (EB) and teratoma formation assays which generated derivatives of all three germ layers. Our results also show that the substitution of knockout serum replacement (KSR) with fetal bovine serum in culture improves the reprogramming efficiency of sheep iPSCs. Generation of sheep iPSCs places sheep on the front lines of large animal preclinical trials and experiments involving modification of animal genomes.

## Introduction

ESC lines, derived from the inner cell mass (ICM) of a blastocyst, can divide indefinitely and are capable of creating all cell types of an adult animal [1]. Isolation of ESC lines from domesticated large animals and ungulate mammals has the potential to enable the precise genetic engineering of livestock for improved production traits, disease resistance and biopharming. Because of their potential use for targeted gene manipulation, isolation of ESCs in livestock may overcome current limitations upon efficient gene transfer by providing an abundance of pluripotent stem cells to be genetically manipulated through the use of conventional recombinant DNA techniques. Unfortunately, genetic alteration in domestic animals has proven to be extremely difficult outside of murine models [2], [3]. The capability to establish pluripotent stem cell lines from large animals is therefore crucial to applications of gene targeting technologies in domestic livestock and non-rodent models.

Domestic sheep are one of the earliest animals to have been domesticated for agricultural purposes. The establishment of sheep ESCs would useful in a number of applications, such as the production of genetically targeted sheep with desired traits and the improvement of somatic cell nuclear transfer efficiency by generation of pluripotent stem cells [4], [5]. More importantly, sheep ESC technology offers an excellent large animal model for human stem cell research because sheep share more phylogenetic characteristics with humans than rodents [6]. Several articles have reported the derivation of ESC-like cells from the ICM of sheep blastocysts [6], [7]. However, the cells derived in these studies do not meet the full criteria to qualify as stable and pluripotent ESCs because they are unable to be maintained in an undifferentiated state beyond two passages in culture. Recently, mouse and human somatic cells have been reprogrammed *in vitro* to induced pluripotent stem cells (iPSCs) by ectopic expression of defined transcription factors. These iPSCs are similar to ESCs in terms of gene expression, pluripotency and epigenetic status, and hold great potential for use in regenerative medicine and *in vitro* disease modeling [8]–[16]. More recently, iPSCs have also been successfully generated from other animals such as monkeys [17], rats [18] and pigs [19]–[21]. In this paper, we report the first generation of iPSCs from sheep somatic cells using a drug-inducible expression system of murine-derived pluripotency factors.

## Materials and Methods

### Cell culture

Sheep fibroblasts used in this report were obtained from a Mongolian sheep fetus at day 40 of gestation. The isolation of sheep fetal fibroblasts (SFFs) was done as previously described [22]. Briefly, an explanted sheep fetus was dissociated manually and then treated with 0.25% trypsin-EDTA (HyClone, Logan, UT, USA). Primary cultures were grown on tissue culture plates coated with 0.1% gelatin (Sigma, St Louis, MO, USA) until the first passage, after which standard tissue culture plates were used. Cells were cultured using Dulbecco's modified Eagle's medium (DMEM, HyClone) supplemented with 10% fetal bovine serum (FBS, Invitrogen, Carlsbad, CA, USA). SFFs were passaged upon reaching 95% confluence with 0.25% trypsin-EDTA and infected with reprogramming virus between passages 3 and 5. 293T cells (ATCC, Manassas, VA, USA) were maintained in the same medium. Sheep iPSCs were maintained in ESC medium composed of Knock-out DMEM (Gibco-BRL, Grand Island, NY) supplemented with 20% Serum Replacement (SR, Gibco) or FBS, 0.1 mM β-mercaptoethanol (Gibco), 1% nonessential amino acids (Hyclone), 2 mM glutamine (Hyclone), and 4 ng/ml hFGF2 (Peprotech, London, UK). Sheep iPSCs were passaged by enzymatic dissociation using 1 mg/ml collagenase IV(Gibco)and maintained on feeder layers of mouse embryonic fibroblasts (MEF) that were mitotically inactivated by mitomycin C (Sigma). Permission to handle all animal samples was granted by Peking University Health Science Center Ethical Committee (PKU20103088).

### Lentivirus production and infection

293T cells were plated at 6×10^6^ cells per 100 mm dish and incubated overnight. The generation and structure of doxycycline-controlled Tet-on-inducible lentiviruses expressing mouse-derived *Oct4*, *Sox2*, *c-Myc* and *Klf4* has been described in previous reports [23], [24]. The c-DNA for GFP was cloned into the EcoRI sites of the same vector backbone (FUW-tetO). Expression of reverse tetracycline transactivator (rtTA) was driven by a constitutively active human ubiquitin C promoter alpha promoter in the FUW lentiviral backbone (FUW-M2rtTA). To produce lentiviral particles used to stably reprogram fibroblasts into iPSCs, 293T cells were transfected with a mixture of viral plasmid and packaging constructs expressing the viral packaging functions and the VSV-G protein with Lipofectamine 2000 (Invitrogen). Viral supernatant was harvested at 24 and 48 hours after transfection. A total of 30 ml of supernatant was typically harvested per virus. Viral supernatant was filtered through a 0.45 µm syringe filter (Millipore, Billerica, MA, USA), and loaded into a Amcion®Ultra-15 Centrifugal Filter (Millipore) for concentration. Viral supernatant was concentrated approximately 100-fold by centrifugation at 6000 rpm for 20 min at 4°C. Viral concentrates were stored at −80°C. Infections were carried out on 35 mm tissue culture plates in 1 ml of medium containing 5 µg/ml polybrene (Sigma) with 5–10 µl of each viral concentrate. SFFs were infected at a density of 5×10^4^ cells/plate, and the medium was replaced 24 hrs after infection. Infection was repeated two times. Cells were subsequently dissociated by trypsin and transferred to plates coated with MEF feeders. To induce reprogramming, culture medium was replaced by ESC medium supplemented with 2 µg/ml doxycycline (Dox).

### RNA isolation and reverse transcription

Total RNA was purified with an RNeasy Mini Kit (Qiagen, Valencia, CA, USA) as per the manufacturer's instructions. Approximately 1 µg of total RNA from each sample was used for Oligo(dT)20 – primed reverse transcription (SuperScript TM III First-Strand Synthesis System for RT-PCR, Invitrogen). PCR products were resolved on (1.5%) agarose gels and visualized by ethidium bromide staining. Images were taken using a gel imaging system (Bio-Rad). The primer sequences employed are displayed in Supplementary Table S1.

### Alkaline phosphatase staining and immunocytochemistry

Alkaline phosphatase (AP) staining was performed using the Alkaline phosphatase kit (Roche Applied Science, Mannheim, Germany) according to the manufacturer's instructions. For immunocytochemistry, cells were fixed with 4% paraformaldehyde for 10 min at room temperature. After washing with PBS, cells were treated with PBS containing 10% normal bovine serum albumin (Sigma) and 0.1% Triton X-100 for 30 min at room temperature, then incubated with primary antibodies at 4°C overnight. Primary antibodies included SSEA-1 (1∶100, Santa Cruz Biotechnology, Santa Cruz, CA, USA), SSEA-3 (1∶100, Santa Cruz), SSEA-4 (1∶100, Chemicon, Temecula, CA, USA), Tra-1-60 (1∶100, Chemicon), Tra-1-81 (1∶100, Chemicon), Nanog (1∶100, Abcam, Cambridge, UK), Oct4 (1∶100, Santa Cruz), Sox2 (1∶100, Chemicon), β III-Tubulin (1∶100, Chemicon), Desmin (1∶100, Santa Cruz), Cytokeratin (1∶100, Santa Cruz). Normal mouse or rabbit serum was used as a negative control. Localization of antigens was visualized with anti-rabbit or anti-mouse IgG secondary antibodies conjugated with fluorescein (Santa Cruz). Nuclei were counterstained with DAPI.

### *In vitro* differentiation

Spontaneous differentiation of sheep iPSCs through EB formation was carried out as previously described [10], [11]. Briefly, sheep iPSCs were cultured in ESC media without hFGF2, hLIF and Dox in non-tissue-culture-treated plates. After 8 days in suspension culture, EBs were transferred to gelatin-coated plates and cultured in differentiation medium for another 8 days.

### Teratoma formation

Sheep iPSCs were harvested by collagenase IV treatment, suspended in PBS and delivered via subcutaneous injection into the dorsal flanks of severe combined immunodeficient (SCID) mice. Eight weeks after injection, tumors were explanted, fixed in 4% paraformaldehyde, embedded in paraffin, and examined histologically using hematoxylin and eosin staining.

### Karyotyping

Sheep iPSCs were prepared for karyotype analysis by incubation in medium containing 0.1 mg/ml colcemid for 5 hours. Cells were trypsinized, resuspended in 0.075 M KCl, incubated at 37°C for 30 min, and fixed in 3∶1 methanol:acetic acid at room temperature for 5 min. Centrifugation and fixing steps were repeated three times. Harvested cells were stained using a standard G-banding technique.

## Results

### Optimization of drug-inducible lentiviral transduction for SFFs

**Figure 1:**
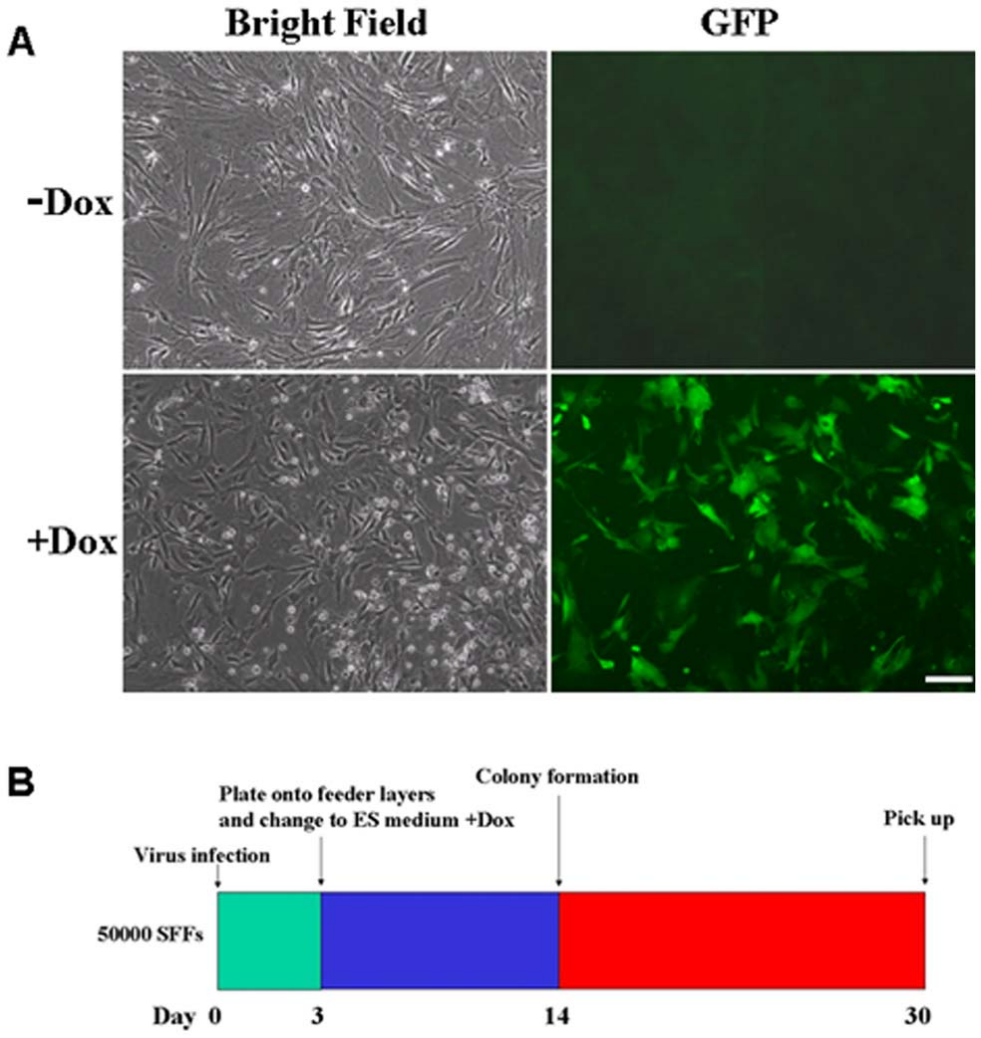
Doxycycline (Dox) controlled Tet-on-inducible lentiviral system for induction of pluripotency in sheep fetal fibroblasts (SFFs). (A) Expression of GFP in SFFs is not observed when Dox is absent from the culture medium. Addition of Dox (2 µg/ml) to the culture medium induces expression of GFP 48 hour after treatment. (B) The time schedule for sheep iPSCs induction. Scale bars:  = 50 µm.

Because induction of iPSCs requires use of lentivirus with high transduction efficiencies, a drug-inducible GFP-expressing lentivirus was added in all transduction experiments to monitor infection efficiency. Over 70% of the SFFs transduced were found to express GFP at high levels 48 hr after Dox addition (Figure 1A).

### Generation of sheep iPSCs

**Figure 2:**
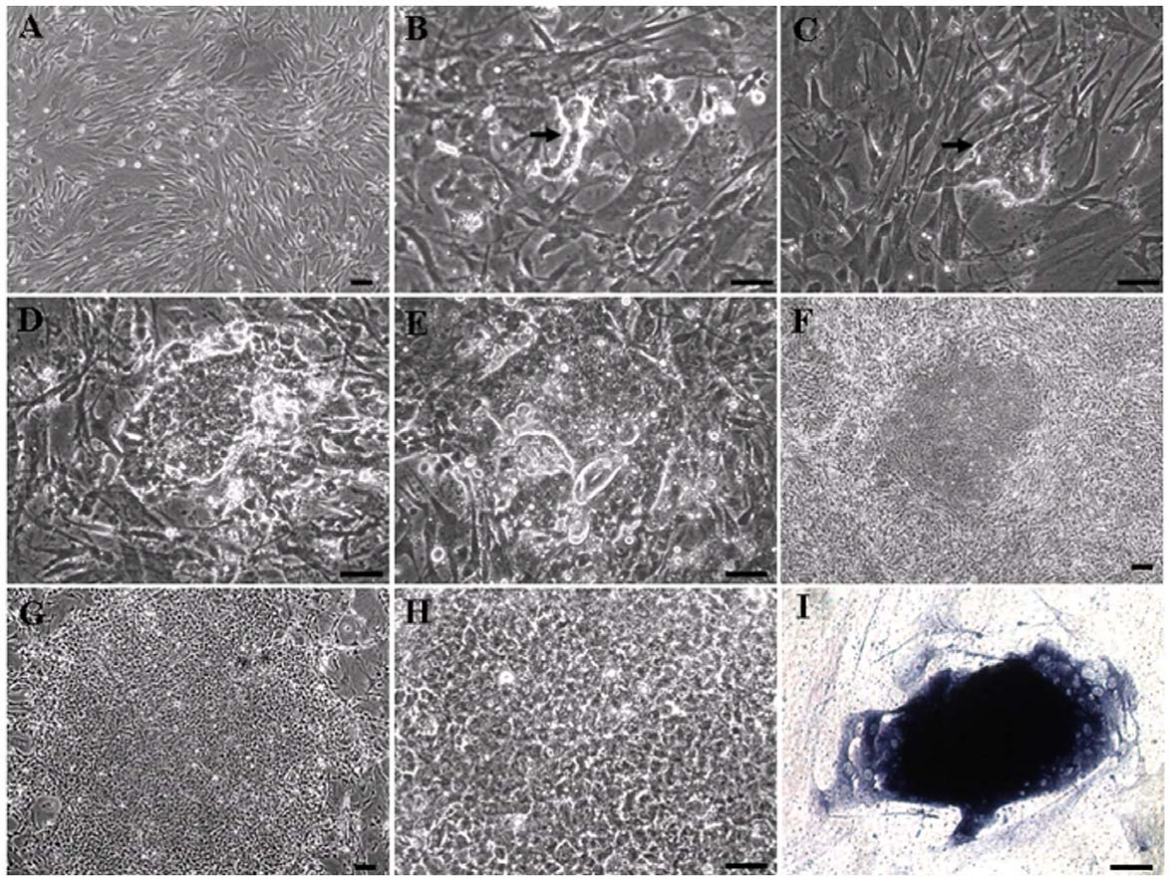
Morphological changes of SFFs undergoing reprogramming to iPSCs. (A) SFFs prior to induction of reprogramming (B) Image of reprogrammed sheep iPSC colony colony day 14, (C) day 16, (D) day 18, (E) day 20, (F) day 25, and (G) day 30 post-transduction. (H) Image of day 30 colony post-transduction with high magnification. (I) Positive AP staining of a colony with typical human ESC morphology. Scale bars:  = 50 µm.

**Figure 3:**
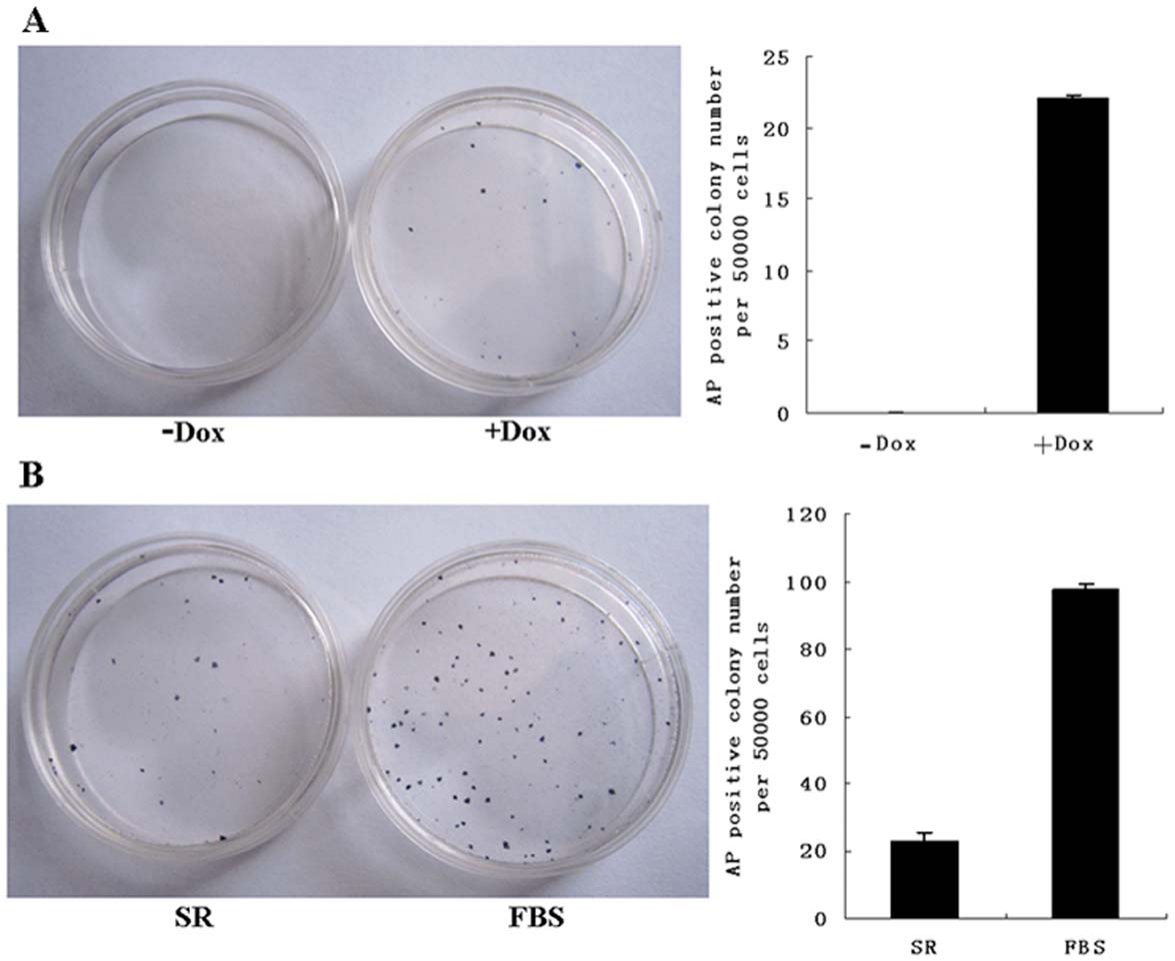
Brightfield microscopy of colonies staining positive for AP following addition of Dox to culture media. Infected SFFs were split into separate dishes and cultured in the presence or absence of Dox. (A) Representative images of Dox negative and Dox positive dishes stained for AP at day 25. (B) Infected SFFs were split into separate dishes and cultured in ESC medium supplemented with Dox and FBS or KSR respectively. Cell cultures were stained for AP day 25 following the initial addition of Dox.

A time table for sheep iPSC induction is shown in Figure 1B. SFFs were transduced at a density of 5×10^4^ cells per 35 mm plate using lentiviruses containing mouse Oct4, Sox2, c-Myc, Klf4, and rtTA genes. Three days after transduction, the cells were trypsinized and plated onto mitomycin C treated feeder cells and cultured using ESC medium supplemented with 2 µg/ml Dox (Figure 2A). Approximately fourteen days later, a few colony-like cell aggregates composed of 5–10 cells each became visible under bright field microscopy (Figure 2B). Over the next week, the size of colonies gradually increased (Figure 2C–E). About day 25 post-transduction, these colonies began to exhibit some human ESC-like features. Colonies became more compact and flatter (Figure 2F). By day 30, ES-cell like colonies had grown very large, and cells within the colonies exhibited morphology similar to that of human ES cells, with a high nucleus-to-cytoplasm ratio and prominent nucleoli (Figure 2G, H). ESC-like colonies were picked and mechanically dissociated into small clumps on top of new feeder layers. AP staining was subsequently used to classify ESC-like colonies as undifferentiated or differentiated. Large ESC-like colonies exhibiting morphology similar to that of human ES cells were found to stain positive for AP (Figure 2I). Cell cultures untreated with Dox were not observed to give rise to any ESC-like colonies (Figure 3A). To test for optimal conditions of sheep iPSC induction and culture, ESC medium containing FBS and KSR were both tested for capacity to maintain cells in an undifferentiated state. We found that the number of AP positive colonies generated from medium supplemented with FBS was significantly greater than in cultures supplemented with KSR (Figure 3B).

### Expression of pluripotent markers in sheep iPSCs

**Figure 4:**
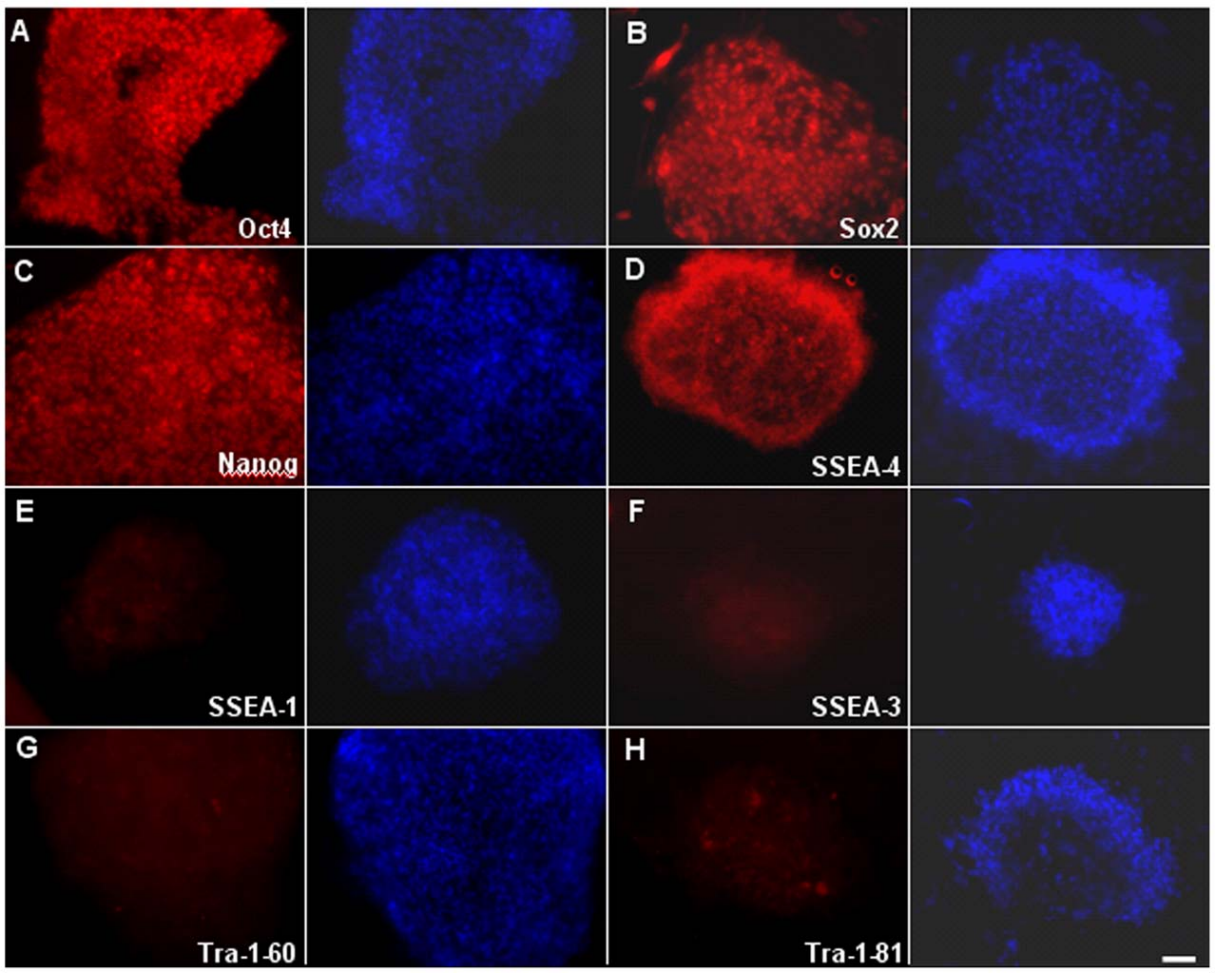
Immunofluorescence staining demonstrates that sheep iPSC colonies are positive for expression of Oct4, Sox2, and Nanog, as well as the surface marker SSEA-4. Colonies were not observed to express SSEA-1, SSEA-3, Tra-1-60, or Tra-1-81. Scale bars:  = 50 µm.

**Figure 5:**
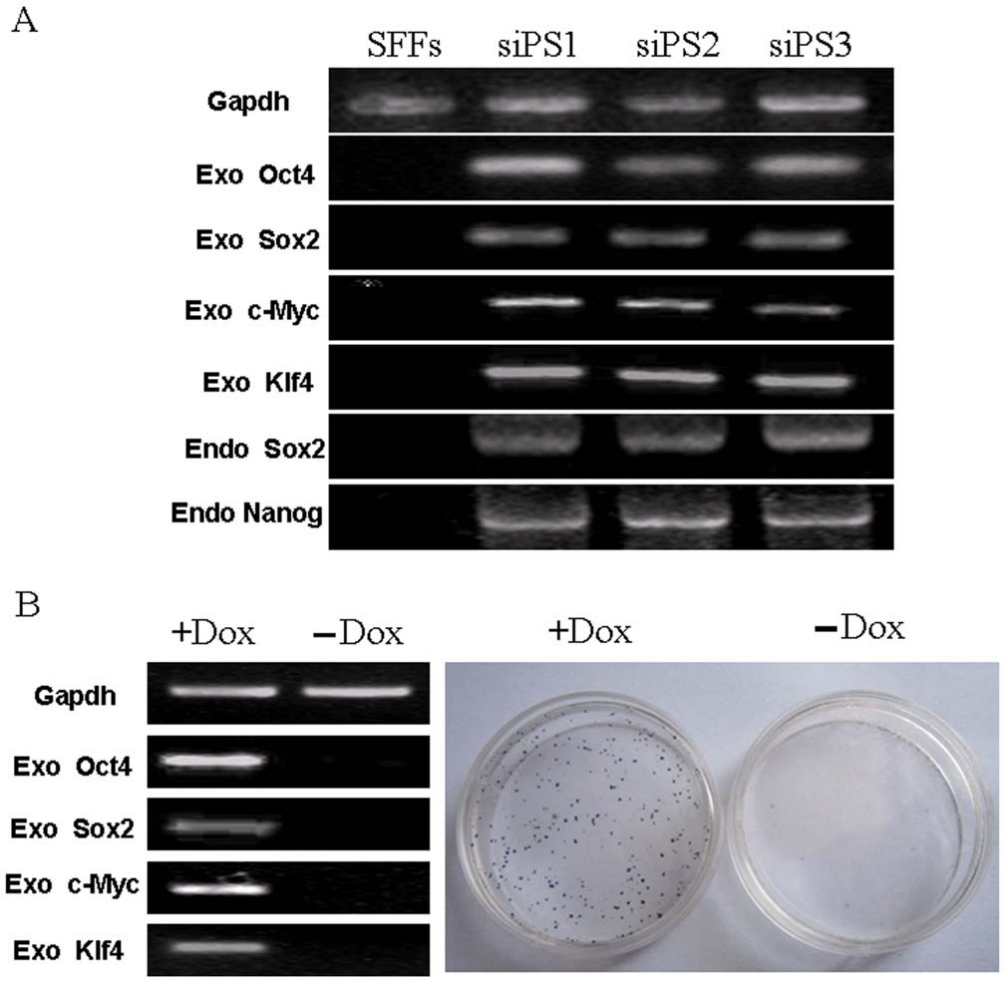
Maintenance of sheep ESC-like colonies is dependent on addition of Dox to culture medium. (A) Three representative sheep iPSC clones (siPS1, siPS2 and siPS3) were selected for analysis of exogenous and endogenous gene expression. (B) Expression of exogenous pluripotency transgenes declined to undetectable levels 15 days after withdrawal of Dox (left panel). The majority of iPSC colonies were observed to turn AP negative following withdrawal of Dox.(right panel).

To confirm that colonies exhibiting ESC-like morphology expressed proteins associated with pluripotent cells, sheep iPSC colonies were stained for a number of surface and intracellular markers associated with undifferentiation in human and mouse cells. Immunofluorescence staining showed that colonies were positive for Oct4, Sox2, Nanog, and SSEA-4, whereas the same colonies were negative for SSEA-1, SSEA-3, Tra-1-60, and Tra-1-81 (Figure 4). Human or mouse ESCs were used as positive controls for immunostaining (Supplementary Figure S1). RT-PCR demonstrated that murine Oct4, Sox2, Klf4 and c-Myc continued to be expressed in sheep iPSC clones through Passage 20, and that endogenous expression of sheep Sox2 and Nanog were activated as well (Figure 5A). Sheep iPSCs could be well maintained beyond 20 passages in ESC medium supplemented with Dox. Following withdrawal of Dox at passage 20, expression of all four transgenes was quickly downregulated, and close to 90% of colonies turned AP negative (Figure 5B).

### *In vitro* differentiation of sheep iPSCs

**Figure 6:**
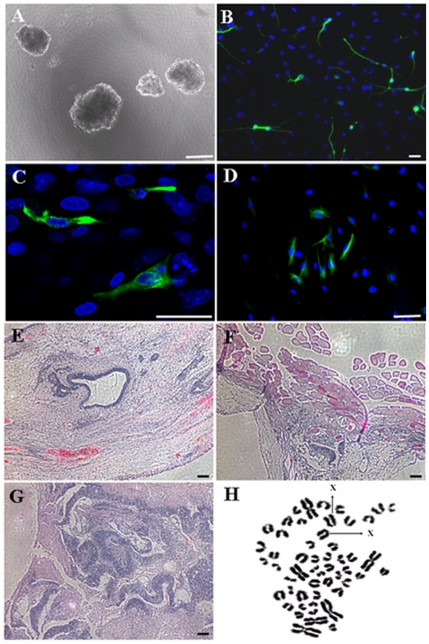
Sheep iPSCs differentiate into derivatives of all three germ layers *in vitro* and *in vivo*. (A) Sheep iPSCs form embryoid bodies in suspension culture following withdrawal of hFGF, hLIF and Dox from culture media. Immunofluorescence staining shows differentiation of sheep iPSCs give rise to cells expressing markers of the three germ layers: (B) β III-Tubulin, (C) Desmin, and (D) Cytokeratin. Hematoxylin and eosin staining of teratomas derived from sheep iPSCs reveals the presence of tissues from all three germ layers: (E) glandular epithelium (endoderm), (F) muscle (mesoderm), and (G) neural epithelium (ectoderm). (H) Sheep iPSCs at passage 15 showed a normal karyotype of 54XX. Scale bars:  = 50 µm.

To assess pluripotency of sheep iPSCs in vitro, we generated EBs from the iPSCs using suspension culture. Sheep iPSCs were observed to form EBs in suspension after 8 days (Figure 6A). EBs were transferred to gelatin-coated plates and cultured for an additional 8 days. Immunofluorescence staining showed that the detected cells were positive for β III-Tubulin (ectoderm), Desmin (mesoderm), and Cytokeratin (endoderm) (Figure 6B-D).

### Teratoma formation and karyotype analysis from sheep iPS cells

To test for *in vivo* pluripotency, sheep iPSCs were transplanted at passage 10 into the subcutaneous flanks of SCID mice. Eight weeks after injection, palpable tumors were observed. Histological examination revealed the presence of derivatives of all three germ layers within the tumor including glandular epithelium, muscle and neural epithelium (Figure 6E-G). Karyotyping was also conducted on iPSCs at passage 15, and revealed a normal karyotype of 54XX (Figure 6H).

## Discussion

Derivation of stable ESC lines has been reported for the mouse [1], [25], hamster [26], rhesus monkey [27], rat [28], [29], and human [30], but validated ESC lines have yet to be established in farm animal species. There are several reasons for the reported difficulties in deriving ESCs from large animals including difficulty in isolating cells at the appropriate stage of embryonic development, suboptimal culture conditions and improper cell passaging methods. Although production of sheep ESC-like cells from early embryos has been reported, these sheep ESC-like cells have not been able to be maintained beyond two passages [6], [7]. Induction of pluripotency in somatic cells by defined factors has been reported in large animals such as pig and dog [19]-[21], [31]. Therefore, iPSC technology offers hope for a practical method by which pluripotent stem cells may be generated from other domestic animals. Here, we generated pluripotent stem cells using drug-inducible expression of defined factors in sheep fetal fibroblasts.

The Doxycycline (Dox) controlled tet-on-inducible system has successfully been applied to generate iPSCs from mouse, human and pig somatic cells [19], [23], [32]. Use of this system is a predictable and highly reproducible platform for iPSC generation and should facilitate the study of early molecular events leading to epigenetic reprogramming of somatic cells [32]. In the present study, we used the Dox controlled tet-on-inducible system to generate and maintain stable sheep ESC-like colonies from fetal fibroblasts. As addition of Dox to culture media was necessary for the induction and maintenance of sheep ESC-like colonies, it is likely that the culture conditions used in this paper are not sufficient to maintain pluripotency in sheep iPSCs alone. Withdrawal of Dox in cultures of stably reprogrammed iPSCs resulted in the silencing of exogenous genes, and differentiation of cells as evidenced by a switch from positive to negative AP staining. To culture sheep iPSCs without use of a drug inducible system, certain growth factors or chemical inhibitors may be required to prevent differentiation. The challenge of optimizing culture conditions for maintenance of pluripotency is the primary reason stable ESC lines have yet to be established in large animals [33], [34]. Cytokines and growth factors that inhibit spontaneous differentiation in mouse and primate ES cell lines such as LIF and bFGF do not inhibit differentiation of pluripotent cells for many large animal ICM and epiblast primary cultures [33]–[36]. In this study, sheep iPSCs were not able to be maintained in an undifferentiated state following withdrawal of Dox even when hLIF and hFGF was added to the medium. Although cells were characterized by a cobble stone patterning with large nucleoli, sheep iPSC colonies generated from the drug-inducible system did not possess a defined, highly refractive edge characteristic of human or mouse ESC and iPSCs. The differences in cell morphology between sheep iPSCs and pluripotent stem cells derived from other animals may be due to imperfect culturing conditions. The maintenance of sheep iPSCs under this drug-inducible system provides a useful platform for studying the role of different cytokines and growth factors in sheep iPSCs self-renewal. This in turn should facilitate the optimization of culture conditions for iPSCs maintenance, and eventual establishment of true ESCs from sheep blastocysts.

While FBS has been used for the establishment of mouse ESC and iPSC lines in a majority of laboratories [11], [25], knockout serum replacement (KSR), which is a defined, serum-free formulation, has been widely used to support the growth of human and primate ESCs and iPSCs in culture [10], [30]. Although detailed information concerning its composition is restricted, KSR does not contain any undefined growth factors or factors that promote differentiation [37]. Our results showed that the replacement of KSR with FBS in culture medium improved the reprogramming efficiency and integrity of sheep iPSCs. These results suggest that serum substrates in FBS may play a vital role in generation of sheep iPSCs, and that these putative factors are not included in KSR.

Cell surface markers provide a powerful tool for characterization and isolation of pluripotent stem cells. Therefore, identification of surface markers expressed on iPSCs is important for pluripotent stem cells. SSEAs including SSEA-1 SSEA-3 SSEA-4, have widely used as cell surface marker to monitor pluripotency of ESCs. Dattena et al. reported that sheep ESCs express SSEA-1, SSEA-3, and SSEA-4 [6]. In this study, sheep iPSCs showed expression of SSEA-4, but not SSEA-1 and SSEA-3. Moreover, our results indicate that sheep iPSCs also lack Tra-1-60 and Tra-1-81, which are characteristic of human ESCs and iPSCs [10], [30]. For future experiments it will be important to compare global gene expression and epigenetic status of pluripotent cell-specific genes between sheep ESCs, iPSCs and somatic cells targeted for reprogramming such as fibroblasts to understand the molecular processes behind induction of pluripotency in sheep cells. Unfortunately, these assays will be difficult to conduct in a sheep model without the release of more comprehensive sheep genomes and commercial sheep gene-chips.

An important proof for the establishment of ungulate pluripotent stem cell lines is demonstration of pluripotency either by differentiation into defined cell types *in vitro* or by teratoma formation *in vivo*. These proofs have been common practice with both mouse and primate ESC and iPSC lines [1], [10], [11], [30]. In the present study, pluripotency of sheep iPSCs was demonstrated by derivation of cells of all three germ layers in EB and teratoma formation assays. However, future work such as chimera formation though injection of sheep iPSCs into developing blastocysts must be performed to truly confirm that these cells are ESC-like.

## Supporting Information

Table S1 Primer sets for PCR reactions.
(DOC)

Figure S1 Positive controls for immunostaining of pluripotency markers Oct4, Sox2, Nanog, SSEA-1, SSEA-3, SSEA-4, Tra-1-60 and Tra-1-81. Scale bars: =50 µm.
(TIF)
