# Supplementary material for: Reprogramming of Sheep Fibroblasts into Pluripotency under a Drug-Inducible Expression of Mouse-Derived Defined Factors
Source: PLoS One. 2011 Jan 6;6(1):e15947. doi: 10.1371/journal.pone.0015947 (PMC3017083; doi:10.1371/journal.pone.0015947)
Supplement: Table S1 — Primer sets for PCR reactions. (DOC) [file pone.0015947.s001.doc]

**Table S1. Primer sets for PCR reactions**

| Genes | Sequence (5' to 3') | Size  (bp) |
| --- | --- | --- |
| For RT-PCR | |  |
| Gapdh | Forward:ACCTTCTGCTGACGCTCCCAT Reverse: GCCATGCCAGTGAGCTTCCCGT | 335 |
| Nanog  (endogenous) | Forward:AGGGTCTGCTACTGAGATGCTCTG Reverse: CAACCACTGGTTTTTCTGCCACCG | 363 |
| Sox2  ( endogenous ) | Forward:AGACTTCACATGTCCCAGCAC Reverse: AAAGAAGTCCAGGATCTCTCATAA | 312 |
| FUW Vector | Forward: CGATAATCAACCTCTGGATTAC |  |
| Exo Oct4 | Reverse:TGTGAGTGATCTGCTGTAGG | 551 |
| Exo Sox2 | Reverse:GGGAAGCG TGTACTTATCCT | 647 |
| Exo c-Myc | Reverse:TCTTCAGAGTCGCTGCTGGT | 654 |
| Exo Klf4 | Reverse:TCTGGTCTGGCAGGAAAGGA | 557 |
