# Supplementary figures and images for: Reprogramming of Sheep Fibroblasts into Pluripotency under a Drug-Inducible Expression of Mouse-Derived Defined Factors
Source: PLoS One. 2011 Jan 6;6(1):e15947. doi: 10.1371/journal.pone.0015947 (PMC3017083; doi:10.1371/journal.pone.0015947)

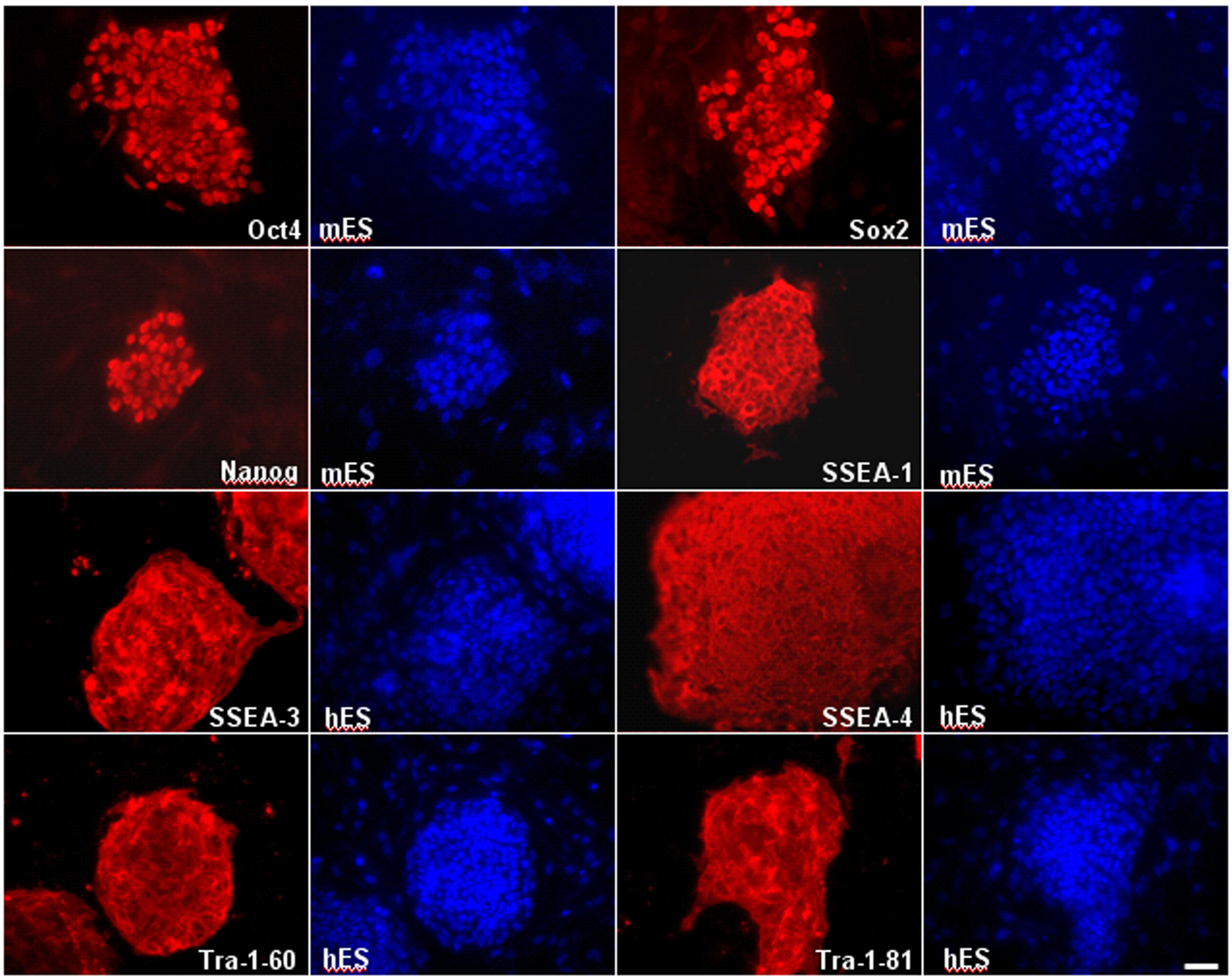

Supplement: Figure S1 — Positive controls for immunostaining of pluripotency markers Oct4, Sox2, Nanog, SSEA-1, SSEA-3, SSEA-4, Tra-1-60 and Tra-1-81. Scale bars: =50 µm. (TIF) [file pone.0015947.s002.tif]
